# Recurrent pain and work disability: a record linkage study

**DOI:** 10.1007/s00420-019-01494-5

**Published:** 2019-11-28

**Authors:** Tea Lallukka, Aapo Hiilamo, Jodi Oakman, Minna Mänty, Olli Pietiläinen, Ossi Rahkonen, Anne Kouvonen, Jaana I. Halonen

**Affiliations:** 1 Finnish Institute of Occupational Health, Helsinki, Finland; 2 Department of Public Health, University of Helsinki, Helsinki, Finland; 3 Centre for Ergonomics, and Human Factors, School of Psychology and Public Health, La Trobe University, Melbourne, VIC Australia; 4 City of Vantaa, Finland; 5 Faculty of Social Sciences, University of Helsinki, Helsinki, Finland; 6 Research Institute of Psychology, SWPS University of Social Sciences and Humanities, Wroclaw, Poland; 7 Administrative Data Research Centre-Northern Ireland (ADRC-NI), Queen’s University Belfast, Belfast, UK; 8 Finnish Institute for Health and Welfare, Helsinki, Finland

**Keywords:** Single site pain, Multisite pain, Recurrence, Sickness absence, Disability pension, Musculoskeletal diseases, Register-based, Occupational cohort

## Abstract

**Purpose:**

We examined the associations between recurrent single- and multisite pain and incident sickness absence (SA) of different lengths and the risk of disability pension (DP).

**Methods:**

The data were derived from the Finnish Helsinki Health Study. Pain measures were recorded for panel 1 in 2000/2 and 2007, and for panel 2 in 2007 and 2012 (altogether 3191 employees). SA data were obtained from the employer’s personnel register and DP events from the Finnish Centre for Pensions. Negative binomial regression models with generalized estimation equations were used to model the incidence of self-certified short- (1–3 days), and medically certified medium- (4–14 days) and long-term (more than 14 days) SA episodes. Cox regression models were fitted for the associations between pain and all-cause DP and competing risk models for DP by diagnostic groups. Social and health-related covariates were adjusted for.

**Results:**

Recurrent pain was associated with short-, medium- and long-term SA. Additionally, recurrent single- and multisite pain increased the risk of long-term SA. Recurrent single or multisite pain was further associated with an increased risk of DP, while a single instance of pain did not increase the risk.

**Conclusions:**

These results suggest that recurrent pain is a robust determinant of subsequent SA and DP risk. Improved understanding of determinants of recurrent pain is needed to inform the development of targeted measures to reduce SA and premature exit from employment.

**Electronic supplementary material:**

The online version of this article (10.1007/s00420-019-01494-5) contains supplementary material, which is available to authorized users.

## Background

General and multisite pain have been associated with a higher risk of sickness absence (SA) and disability pension (DP), but many studies have been either cross-sectional, have used only one measurement of pain over time, or assessed self-reported work disability (de Fernandes and Burdorf 2016; Saastamoinen et al. 2012; Neupane et al. 2011; Kääriä et al. 2012; Andersen et al. 2011; Burdorf and Jansen 2006). While multisite pain is a stronger predictor of sickness absence than single site pain (de Fernandes and Burdorf 2016; Haukka et al. 2015; Neupane et al. 2011), longitudinal research examining recurrent single or multisite pain as predictors of SA and DP are rare. A recent study in the British Whitehall II cohort showed that recurrent pain is a predictor of premature exit from the labour market due to health reasons, whilst those reporting a single instance of pain were not at risk (Lallukka et al. 2018). The follow-up period was long, but all exit routes from paid employment were self-reported, and SA was not differentiated from DP. Moreover, no diagnostic groups could be distinguished, i.e., any health-related exit, short and long-term and all diagnoses were merged. Thus, it is not known, to what extent recurrent pain predicts exit from paid employment due to both musculoskeletal diseases and mental disorders. A key limitation of the previous analysis was the reliance on back pain as the only indicator of pain. However, in most cases pain exists across multiple sites, with recurrence a common issue (Neupane et al. 2017). Previous studies suggest that multisite pain predicts both SA and DP (Haukka et al. 2013, 2015). However, to the best of our knowledge, recurrent multisite pain as a predictor of work disability across a range of severity measures has not been previously examined.

To address these gaps, the current study examines the association between recurrent single and multisite pain, and work disability using register-based data, while controlling for social and health-related factors (Hemingway et al. 1997; Dionne 2010; Schneider et al. 2005; Kaila-Kangas et al. 2009). Outcomes include short, medium length, and long SA, and any full-time, part-time, fixed-term or permanent DP. Additionally, two key diagnostic groups of DP are included in the current analysis: musculoskeletal diseases and mental disorders.

## Methods

The data were derived from the ongoing Helsinki Health Study (HHS), a longitudinal occupational cohort of employees of the City of Helsinki, Finland, aged 40–60 years at baseline in 2000–2002 (Lahelma et al. 2013). The City of Helsinki is the largest employer in Finland, with hundreds of different occupational titles represented. Data comprised repeated mailed surveys (response rates ranging from 67 to 83%), linked to administrative employer’s personnel register data on SA and national pension register, for those participants who had provided written informed consent for both linkages (6487 persons). Previous analysis of non-respondents and those who had provided consent support with the data being broadly representative of the target population. However, some groups are less likely to participate such as those with long SA episodes, lower socio-economic status, men, and those in the youngest age group (Lahelma et al. 2013; Laaksonen et al. 2008). However, it is unlikely that the non-response distort or bias the analysis for the work disability outcomes (Martikainen et al. 2007).

Three consecutive surveys conducted in 2000-2 (phase 1), 2007 (phase 2) and 2012 (phase 3) and register-based follow-up data after the survey participation on SA episodes and DP awards from 2007 to 2016 were used for the current study. Inclusion criteria were as follows: participation in two consecutive surveys either in phase 1 and 2 (panel 1) or phase 2 and 3 (panel 2), and provision of consent for data linkage. Exclusion criteria were: individuals who were aged 63 (disability pension is not granted after that age) or with a pension awarded before the 2007 survey, or without continuous employment at the time of the survey (in total 2076 persons were excluded). In addition, employees with missing data on the two measures of pain were excluded (226 persons) resulting in 3191 persons (5109 observations for the SA and 5149 records for the DP analyses) [see Online Resource Fig. 1 for complete sample selection].

### Types of recurrent pain

**Fig. 1:**
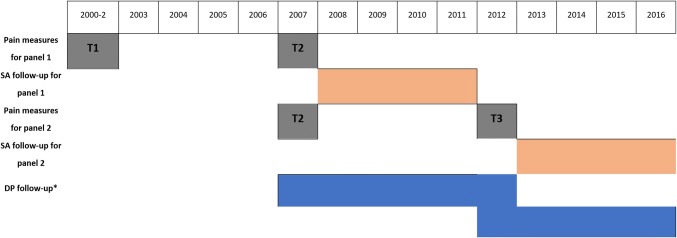
Illustration of the study design. *Time varying covariates from T2 to T3 (from 2012, i.e., T3 for the other covariates). For those who did not respond the T3 survey the follow-up was ended in 2012, as for them, it is not possible to address if their pain or covariates changed

Types of recurrent pain were categorised according to participants’ pain reporting in two consecutive surveys which took place 5–7 years apart (see Fig. 1). Individuals were asked whether they were currently experiencing any acute/subacute or chronic pain. Secondly, they were asked to specify the locations of their pain (head/face, neck/shoulders, low back, lower limbs, upper limbs, stomach and a self-reported location). Based on responses to these questions, a categorical multisite pain measure was created: no pain, single-site and multisite pain (two or more pain locations). Those participants who reported pain but did not report pain locations were considered missing cases. In addition, participants not responding to the question related to the current experience of any pain while reporting no pain areas were also set as missing cases. Based on responses to the two subsequent surveys, nine types of recurrent pain were created: 1 “No past or current pain (the reference group)”, 2 “Only current single-site pain”, 3 “Only current multisite pain”, 4 “Only past single-site pain”, 5 “Recurrent single-site pain”, 6 “Past single-site, current multisite pain”, 7 “Only past multisite pain”, 8 “Past multisite pain, current single-site”, 9 “Recurrent multisite pain”.

### Sickness absence and disability pension outcomes

SA and DP records were used to identify work disability. Three outcomes were used for SA: the number of (1) short-term SA episodes (1–3 days), (2) medium-term SA episodes (4–14 days requiring medical certificate), and (3) long-term SA episodes (more than 2 weeks requiring medical certificate) during the follow-up period. The follow-up periods were for panel 1 from 2008 (i.e., the year following the second survey year) to 2011 (or until the end of the work contract with the City or death), and for panel 2 from 2013 to 2016 (or until the end of the work contract or death). The mean number of sickness absence spells by the types of recurrent pain is shown in Online Resource Table 1.

For any DP (fixed-term, permanent full-time or part-time) the follow-up time commenced from the date the participant returned the phase 2 (in 2007) mailed survey and continued until the end of 2012 or the end of 2016, other form of pension, reaching 63 years of age (the age after which disability pensions are no longer granted), or death. DP follow-up terminated at the end of the year 2012 if the participant was a non-respondent in the 2012 survey (phase 3). This restriction was made as for those not responding at phase 3, no information was recorded as to whether or not any of their covariates changed during the mid-period of the follow-up. The mean follow-up time was 6.3 years for the 258 all-cause DP awards (of them 123 due to diseases of the musculoskeletal system and connective tissue, and 71 due to mental, behavioural and neurodevelopmental disorders, and the rest for heterogeneous causes). DP due to musculoskeletal diseases and mental disorders, the major diagnostic groups (Finnish Centre for Pensions (ETK) and The Social Insurance Institution of Finland (SII) 2016), were used for additional analyses. The number of DP events by the types of recurrent pain (main exposure) and the crude rates are shown in Online Resource Table 2.

### Covariates

We included covariates that are potentially linked to work disability and pain (Oakman et al. 2017a, b; Haukka et al. 2012, 2014; Saastamoinen et al. 2005). All models were adjusted for gender. Age was controlled as a continuous variable in the SA analysis, whereas in the Cox models we used age as a timescale. Marital status was defined as married or cohabiting vs. never married, divorced or widowed. Occupational class was measured by occupational title and grouped into (1) managers and professionals, (2) semi-professionals, (3) routine non-manuals and (4) manual workers. We also included two subjective measures of working conditions. Employees were asked to rate how they perceived their mental and physical working environment. Those responding as having very demanding mental work were classified as working in mentally demanding (adverse) working conditions; those who reported very or somewhat strenuous physical work were coded as having strenuous (adverse) physical working conditions. Differences in the cut-off for the dichotomization between mental and physical work were due to different distributions of the responses for these items. Thus, adverse mental working conditions were more commonly reported compared to physical conditions. The variable describing shift work was classified into night or other shift work vs. regular day shifts, and part-time work (less than 30 h a week) vs. full-time work.

Obesity (body mass index ≥ 30 vs. others), was included as it has been shown to be linked to both pain (Shiri et al. 2013, 2014, 2019) and work disability (Roos et al. 2013; Korpela et al. 2013), hence we considered it mainly as a confounding factor. Other health-related measures (potential confounders) included: self-reported long-standing illness and self-reported physician diagnosed chronic illnesses that were potentially pain-related (arthrosis, rheumatoid disease, migraine and cancer). A measure of common mental disorders (CMD) was also included (general health questionnaire GHQ-12 score of 3 or more) (Goldberg 1972).

### Statistical analysis

Statistical analyses were conducted in three steps. First, descriptive statistics indicating the distribution of the covariates according to the nine types of recurrent pain were provided. Prevalence of specific pain locations reported and SA and DP episodes by the types of recurrent pain were also explored.

Second, the risks of SA episodes of different length were modelled using negative binomial regression models. The number of SA episodes was used as a count outcome variable. Negative binomial distribution was chosen over Poisson due to overdispersion in the outcome variables. To account for the different exposure time to SA, the time from the beginning of SA follow-up to the end date of the job contract with the city of Helsinki was used as the exposure time. Generalized estimation equations (GEE) were used to consider that employees were potentially present in both panels. GEE is designed for repeated measures data and the method handles the within-individual correlation through the utilisation of a correlation matrix (Liang and Scott 1986). The coefficients were transformed into incidence rate ratios (IRRs with 95% confidence intervals (CI)), that is the factor by which the expected incident rate of SA spells (potentially recurrent event) is increased between the reference and exposed group(s).

Third, time to the first DP event was modelled using Cox proportional hazard model with left truncated data and age as a time scale (i.e., delayed entry, because older participants were excluded if they had previously received a DP) (Cleves et al. 2008; Lamarca et al. 1998). Follow-up for DP began the day after returning the 2007 survey and lasted until the end of 2016, or end of 2012 if the participant was non-respondent in the 2012 survey (because for them we lacked information whether or not their pain and covariates changed). For those employees present also in the panel 2 and who were not awarded DP or experienced a censoring event before the 2012 (phase 3) survey, we used time-varying covariates from panels 1 and 2. Thus, a participant responding to all three surveys could move from one longitudinal pain group to another, or/and have a change in any of the covariates (except gender) during the DP follow-up. The acceptability of the proportional assumption was assessed based on Schoenfeld residuals and was satisfied in all used models (see below for details). In models studying the effect on cause-specific DP, DPs due to other reasons were used as competing risks. Results are presented as hazard ratios (HR) with 95% CI.

We did not find any systematic interaction effects of the type of multisite pain and gender (or the four categories of occupational class) on work disability outcomes. Thus, the results are not shown for men and women separately. Furthermore, the sample and the studied types of multisite pain were not large enough to conduct stratified models by specific occupations.

We first ran model 1 adjusting for age (only in SA analyses), gender and the panel (1 or 2). Subsequent model 2 was simultaneously adjusted for age (only SA), gender, panel, occupational social class, long-standing illness, pain-related illness, working conditions, part-time work, marital status, common mental disorders, shift work and obesity. Around 6% of the observations had some missing items on the covariates used in model 2 that were handled by deleting observations with missing items. Therefore, analytical sample was slightly smaller in the adjusted models: 3087 participants (4788 observations) were included in model 2 of SA and 3092 (4827 observations) in model 2 of DP. The number of observations in models 1 and 2 was slightly smaller for SA since participants were required to be employed by and, therefore, in the personnel SA register of the City of Helsinki also in phase 3. For the DP analyses, we had data from national administrative records also for those who changed employers after the 2007 survey. Results are presented as figures. All analyses were conducted using Stata 15.

## Results

Distributions of the types of recurrent pain in total and by gender in 2007 are shown in Table 1. Around 44% of participants reported no pain in the current or past survey (54% of 575 men, 42% of 2616 women). Approximately, a fourth of all participants reported having single-site pain; either current only (9%), past only (10%) or recurrent (5%). The remaining participants comprised mainly those reporting past single site and current multisite (7%), only current multisite (8%), and recurrent multisite (9%). Very few individuals moved from having multisite pain in the past to single-site pain only (3%) or to no current pain (4%).

**Table 1:** Descriptive statistics of the study population by types of recurrent single and multisite pain in panel 1 (2007)

Types of recurrent pain	Sex			Sample size	Sex difference for nominal data (Chi square)
	Men (575 persons)	Women (2616 persons)	Total		
	Col %	Col %	Col %		*p* value
No past or current pain	54	42	44	1408	50.254
Only current single-site pain	10	9	9	294	0.000
Only current multisite pain	5	8	8	249	
Only past single-site pain	11	10	10	310	
Recurrent single-site pain	3	5	5	154	
Past single-site, current multisite pain	4	8	7	236	
Only past multisite pain	5	4	4	142	
Past multisite pain, current single-site	2	4	3	109	
Recurrent multisite pain	6	10	9	289	
Total	100	100	100	3191	

The Helsinki Health Study

The prevalence of long-standing and pain-related illnesses (arthrosis, rheumatoid disease, migraine and cancer) was highest in the recurrent pain groups (Table 2). The prevalence of common mental disorders (CMDs) and obesity were lowest among participants who reported no current or past pain, while the prevalence figures were higher among participants with single or multisite recurrent or current pain. Furthermore, those with recurrent multisite pain reported often mentally demanding and physically strenuous working conditions. Regarding the location of pain, individuals with recurrent multisite pain had the highest prevalence of current neck or shoulder pain (84%) (see Online Resource Table 3 and Online Resource Table 4). In contrast, for those with only current single-site pain, the lower limbs were the most commonly reported location (30%). Employees with some form of recurrent pain had the highest mean number of SA spells of any length and the highest crude rate of DP events (see Online Resource Tables 1 and 2).

**Table 2:** Descriptive statistics of the study population by types of recurrent single and multisite pain in panel 1 (2007)

	Types of recurrent single and multisite pain										Total*
	No past or current pain (*n* = 1408)	Only current single-site pain (*n* = 294)	Only current multisite pain (*n* = 249)	Only past single-site pain (*n* = 310)	Recurrent single-site pain (*n* = 154)	Past single-site, current multisite pain (*n* = 236)	Only past multisite pain (*n* = 142)	Past multisite pain, current single-site (*n* = 109)	Recurrent multisite pain (*n* = 289)	Total (*n* = 3191)	
	Col %	Col %	Col %	Col %	Col %	Col %	Col %	Col %	Col %	Col %	*N*
Sex											
Men	22	19	11	20	13	11	19	12	11	18	575
Women	78	81	89	80	87	89	81	88	89	82	2616
Occupational status											
Managers and professional	38	38	27	35	34	22	30	28	21	33	1065
Semi-professionals	24	24	24	25	19	24	24	26	16	23	746
Routine non-manual workers	29	28	35	34	38	42	32	35	46	33	1050
Manual workers	9	10	13	7	8	11	15	12	17	10	326
Marital status											
Married or cohabiting	70	71	70	65	76	67	65	76	68	69	2216
Never married, divorced or widowed	30	29	30	35	24	32	35	24	32	30	969
Pain-related illness (rheumatoid disease, cancer, arthrosis or migraine)	26	41	45	28	46	55	35	49	57	36	1157
Long-standing illness	33	51	64	38	58	64	39	61	68	46	1453
Common mental disorders	14	30	31	17	28	41	28	24	44	23	747
Obesity	13	16	24	15	21	19	20	17	22	17	532
Physically strenuous working environment	24	26	36	25	29	42	35	36	44	29	936
Mentally strenuous working environment	9	13	18	11	11	14	12	17	18	12	375
Shift work or night work	15	16	20	18	17	19	19	21	15	16	525

*Columns not sum to total *n/100%* if missing values

**Fig. 2:**
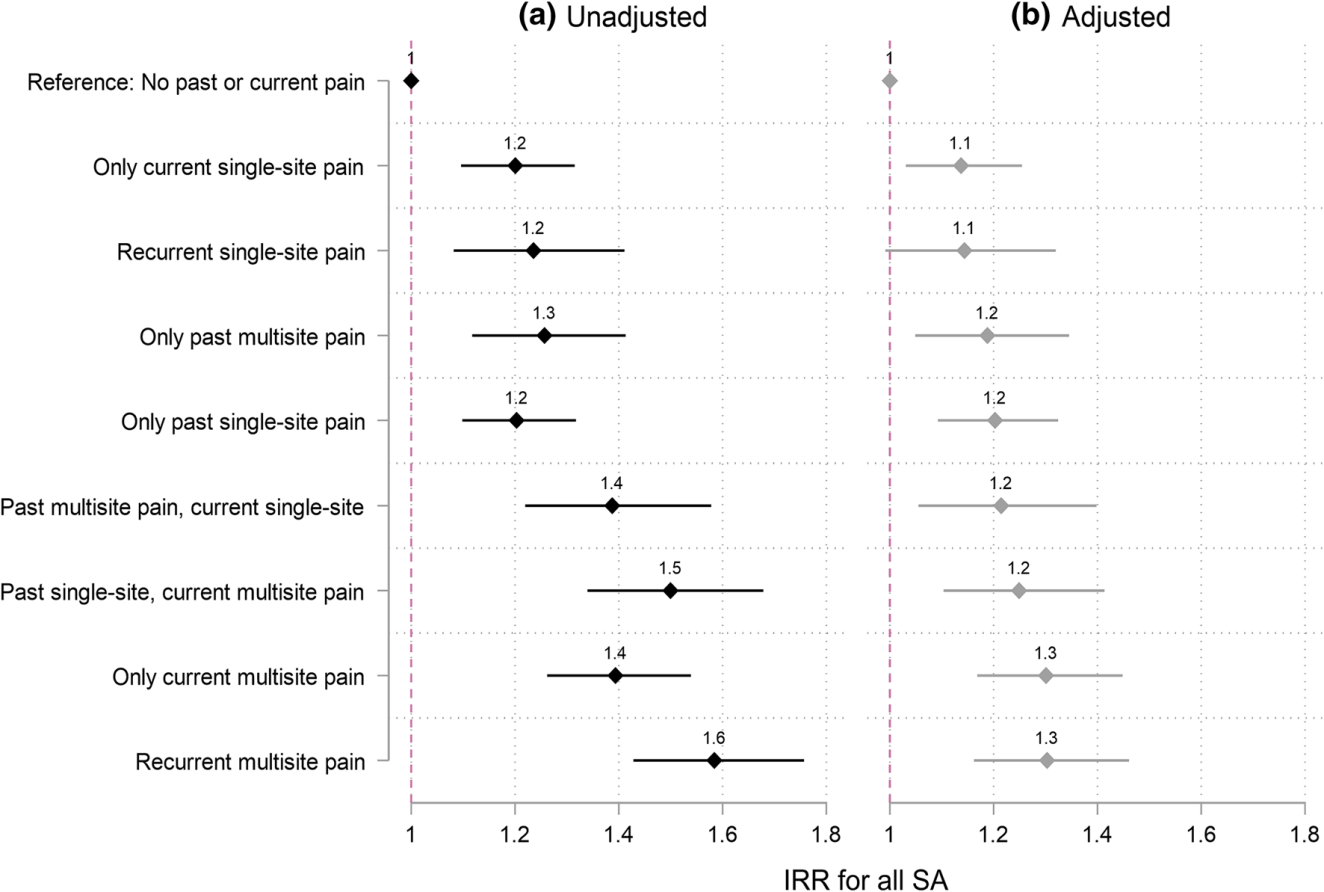
Incident rate ratios (IRR) for the association between types of recurrent pain and all sickness absence (SA) episodes. Reference: no past or current pain. **a** Unadjusted model = adjusted for gender, measurement period and age. **b** Adjusted model = additionally adjusted for occupational social class, long-standing illness, pain-related illness, working conditions, part-time work, marital status, common mental disorders, shift work and obesity

**Fig. 3:**
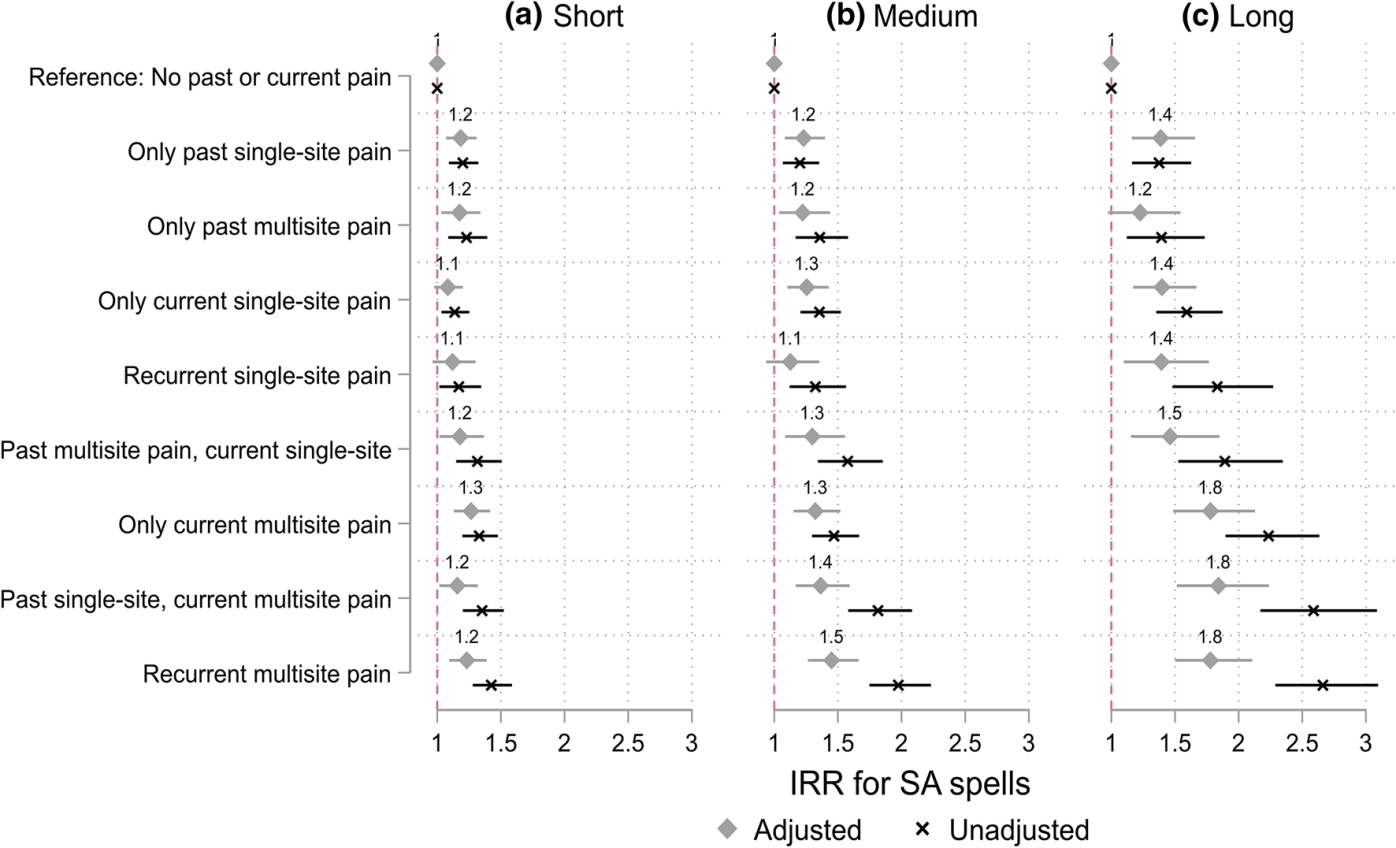
Incident rate ratios (IRR) for the association between types of recurrent pain and **a** short-term (1–3 days), **b** medium (4–14 days), **c** long-term (more than 2 weeks) sickness absence (SA) episodes. Reference: no past or current pain. Unadjusted model = adjusted for gender, measurement period and age. Adjusted model = additionally adjusted for occupational social class, long-standing illness, pain-related illness, working conditions, part-time work, marital status, common mental disorders, shift work and obesity

The associations between the course of pain and any SA or SA of different lengths are shown in the Figs. 2 and 3, respectively. After adjusting for gender, measurement period and age, all pain groups were associated with any SA (Fig. 2). After full adjustment, the associations somewhat attenuated but remained for all groups except recurrent single-site pain.

For short-term SA, all pain groups showed slightly increased risk after adjusting for gender, measurement period and age. The associations remained after adjusting for covariates, except for those reporting only current single-site pain and recurrent single-site pain (Fig. 3). For the other groups, the IRRs in model 1 ranged between 1.2 and 1.4, and the adjusted IRRs between 1.2 and 1.3.

All groups of pain increased the risk of medium-term SA (Fig. 3). The strongest association was found between recurrent multisite pain and medium-term SA (IRR 2.0 [95% confidence interval 1.7–2.2]), but as for the short-term SA, the IRR attenuated after controlling for social and health-related covariates (IRR 1.5 [1.3–1.7]). However, all associations remained, except for the recurrent single-site pain.

All groups of pain similarly showed an increased risk of a long-term SA, with the strongest associations for the recurrent multisite pain (IRR 2.7 [2.3–3.1]). After full adjustment, the IRRs attenuated and were of similar level (IRR 1.8) for recurrent multisite pain, only current multisite pain, and past single-site pain current multisite pain. In addition, all of the other associations remained after full adjustment, except the association between only past multisite pain and long-term SA was not confirmed.

**Fig. 4:**
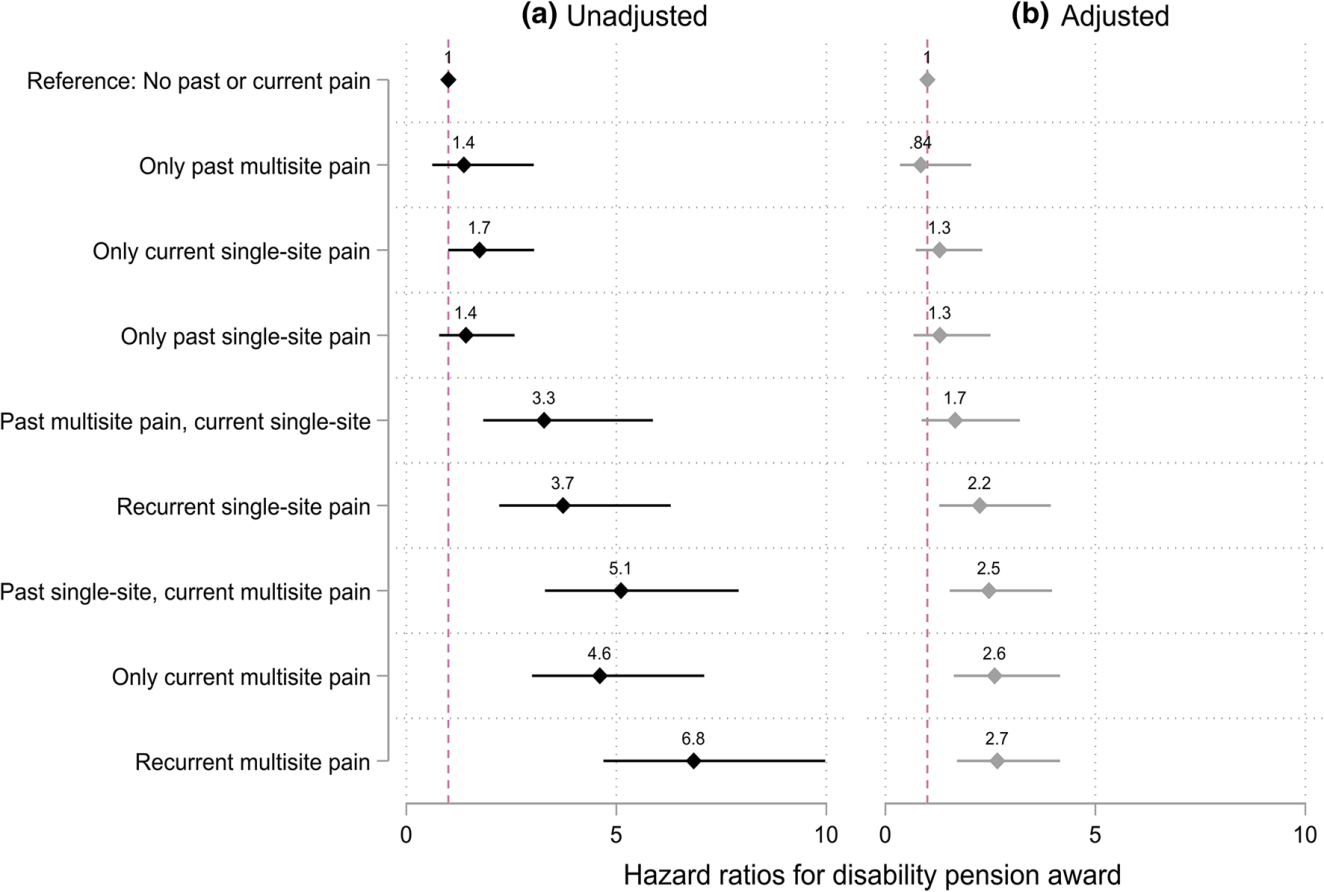
Hazard ratios for the association between types of recurrent pain and all-cause disability pension award. Reference: no past or current pain. **a** Unadjusted model = adjusted for gender, measurement period. **b** Adjusted model = additionally adjusted for occupational social class, long-standing illness, pain-related illness, working conditions, part-time work, marital status, common mental disorders, shift work and obesity

The risk of disability pension is shown in Fig. 4. After adjusting for gender and measurement period, recurrent single site (a hazard ratio, HR, of 3.7 [2.2–6.3]) and recurrent multisite pain (HR 6.8 [4.7–10]) were strongly associated with the risk of DP. Following a full adjustment, the risk estimates were notably attenuated, but remained significant (2.2 [1.3–3.9] and 2.7 [1.7–4.2], respectively). No risk could be confirmed for only past multisite, only current and only past single-site pain, and past multisite current single-site pain after full adjustments.

Analyses using diagnostic groups of DP as outcomes (see Online Resource Fig. 2) showed that employees with recurrent pain and those with only current multisite pain had the highest risk of DP due to musculoskeletal diseases. Furthermore, before adjusting for health and work-related characteristics, these types of recurrent multisite pain were associated with DP due to mental disorders, but the associations were reduced after full adjustments.

## Discussion

### Main findings

We examined the contribution of recurrent single and multisite pain to the severity of the work disability. Several key findings were identified. First, a range of characteristics was identified which differentiate those with recurrent pain from those with no pain (e.g. CMD and obesity). This information can be used in the development of prevention strategies. Second, recurrent single and multisite pain were important predictors of SA of all lengths, and DP. Third, the more extended the absence from work, the stronger were the associations. Fourth, adjustment for health- and work-related factors attenuated the associations between recurrent pain and work disability. Finally, DP related to recurrent pain was largely attributable to disability due to musculoskeletal diseases; in addition, some links between pain and work exit may be attributed to the presence of mental disorders.

### Previous studies

This study supports the significant influence of recurrent pain on early health-related exit from paid employment (Lallukka et al. 2018). A key strength of the current study is the focus on multisite recurrent pain linked with register-based outcome data. Moreover, distinguishing between work disability outcomes of different severity, namely SA of different lengths and DP, provides new insights into this area. While previous research has identified that pain is a predictor of early exit from paid employment (Neupane et al. 2011, 2016; Haukka et al. 2013, 2015; Saastamoinen et al. 2012; Kääriä et al. 2012; Andersen et al. 2011; Saastamoinen et al. 2009; Burdorf and Jansen 2006), this study extends these findings by providing more specific insights into which groups of individuals with pain are at the highest risk of early exit, and the key reasons determining their health-related exit. Multisite pain and those with recurrent pain were at a higher risk of early exit from work; therefore, workplace prevention strategies to reduce the incidence of pain are important. However, given the recurrent nature of pain, another aspect of workplace management is the provision of appropriate support to enable those with pain (both multisite and single site) to continue working. Workplace accommodations have been identified as an important part of managing musculoskeletal conditions at work and prevention of work disability (Oakman et al. 2016a, b, 2017a, b).

The strongest risks were observed for those with multisite and single-site recurrent pain, whereas a single past episode of pain did not notably increase the risk of work disability. While a measurement of pain on one occasion only appears to be a relatively poor indicator of the contribution of pain to work disability, it is important to note that its measurement is needed for early detection of people with pain, in efforts to prevent pain from becoming chronic, which would increase the risk of health-related exit. In addition, an important note is that recurrent single-site pain could lead to the development of multisite pain. Any dichotomous outcome merging any work disability and all health-related exits (SA and DP) is also likely to inadequately fully capture the links between pain and work participation. As expected, the associations between pain and work disability were stronger the longer the length of absence from work. However, recurrent pain contributed to work disability of all lengths, including short SA episodes. Although self-reported pain on its own is not a sufficient cause for a DP award, it is a potential trigger and a potential indicator of other underlying diseases.

## Methodological considerations

### Limitations

Several limitations in the study require acknowledgement. First, the cause of the SA was unknown and analysis on diagnostic groups could only be undertaken for DP, where the ICD-10 codes were available. It is reasonable to assume that the results would be similar for SA as it precedes DP. Second, the pain data were self-reported; however, pain is a subjective condition and as such this is the most effective way to collect information on a person’s condition (Dionne 2010). Another limitation to self-reported pain is that some psychosocial factors may influence pain reporting (Schneider et al. 2005), although those, such as job demands, did not contribute to the associations in these data. Additionally, no specific data relating to the cause of pain or its severity were available. We measured pain at seven locations, but some other studies have used data with more pain sites (Haukka et al. 2013). However, key locations were included such as pain in the upper and lower extremities and back.

Third, the time period between surveys was relatively long, which may mean some information was missed in relation to pain, and unmeasured confounding cannot be ruled out. Fourth, as we adjusted for several pain-related illnesses, longstanding illnesses and mental ill-health, this might be considered as overadjustment. As diagnosed illness is a prerequisite for a grant of long-term SA or DP, including these in the full model could have introduced bias in our estimates (Schisterman et al. 2009). Adjustment theoretically means that we focus on the ‘independent’ effect of pain on the outcomes after ruling out the effects of illnesses, but such a situation is not possible in real life. Mental ill-health could also be considered being in the causal path between the exposure (pain) and our outcomes. However, in this design, it is not possible to distinguish between causal order regards to the confounders (or mediators) and exposures, since they were measured at the same time. For this reason, we report the gender and measurement period (and age) adjusted results first, which show the associations between pain and the outcomes prior to adding the potentially questionable health-related variables in the models. Nonetheless, without considering health at baseline, the study would remain relatively descriptive with some unanswered questions, such as does pain add to the effect that having an illness has on the risk of SA and DP. We have shown earlier that people who have cross-sectionally measured pain and either report or do not report longstanding illness, have an increased risk of DP, but the risks are higher for those with both pain and longstanding illness (Saastamoinen et al. 2012). Therefore, pain could also precede illnesses, which could explain why it remains a predictor of SA and DP even after full adjustments, justifying our decision to show both unadjusted and adjusted associations. Finally, we had a highly female-dominated cohort. This highlights that care with generalisation of the results must be taken since women are more likely to report pain in comparison to men (Schneider et al. 2006). Moreover, due to differences in welfare regimes; reimbursement for sick leave and entitlement for DP, it is possible that the results are different in different contexts such as in other European countries, USA or Canada. However, in general, pain is a common problem worldwide, and for example years lived with disability are strongly connected to low back pain globally (GBD 2017 Disease and Injury Incidence and Prevalence Collaborators 2018).

### Strengths

This study also had several strengths. First, we had complete register-based data for outcomes which helped to reduce common method bias, where people could self-report both high levels of exposure (pain) and the outcome. An additional strength is the inclusion of both short- and long-term work disability, to consider the severity of work disability, and to examine whether recurrent single and multisite pain similarly contributed to both short- and long-term SA as well as DP. The register-based outcomes also assist with completion of the follow-up, i.e., attrition is minimal in comparison to survey-based follow-up, where loss to follow-up is often a significant issue. The diagnostic groups related to DP helped confirm whether the previously demonstrated association between recurrent pain and health-related exit (Lallukka et al. 2018) is mainly due to musculoskeletal diseases or mental disorders. Furthermore, the repeated measures from the same participants enabled the examination of recurrent pain. We could also distinguish between repeated single site and multisite pain. A further strength was the large cohort, which included employees from a wide range of occupations. Additionally, a number of health and work-related covariates was included. Such information is not available in the registers; therefore, linking rich survey data with high-quality register data provides a more comprehensive set of information than would be obtained using only one source.

## Conclusions

Measuring recurrent pain and the number of pain sites may offer an effective way to estimate the risk of SA and DP, as compared to pain measured only at a single time point. Pain is an important contributor to work disability and early identification is important so that more effective prevention strategies can be developed. A key aspect of effective prevention will require employers to engage with their workers so that their needs can be identified (Oakman et al. 2016a, b, 2017a, b). Workplace interventions to enable people with recurrent pain to continue working are an important part of prevention strategies, and relevant to an individual, which will vary depending on their condition and the work being undertaken.

## Electronic supplementary material

Below is the link to the electronic supplementary material.

Supplementary material 1 (DOCX 815 kb)
