# Supplementary material for: Recurrent pain and work disability: a record linkage study
Source: Int Arch Occup Environ Health. 2019 Nov 28;93(4):421–32. doi: 10.1007/s00420-019-01494-5 (PMC7118055; doi:10.1007/s00420-019-01494-5)
Supplement: Supplementary file 1 — Supplementary material 1 (DOCX 815 kb) [file 420_2019_1494_MOESM1_ESM.docx]

Online Resources

**Recurrent pain and work disability: a record linkage study**

T Lallukka* (1,2), A Hiilamo* (1), J Oakman (3), M Mänty (2,4), O Pietiläinen (2), O Rahkonen (2), A Kouvonen (5-7), JI Halonen (1,8) *shared 1^st^ authorship

1) Finnish Institute of Occupational Health, Helsinki, Finland

2) Department of Public Health, University of Helsinki, Helsinki, Finland

3) Centre for Ergonomics, and Human Factors, School of Psychology and Public Health, La Trobe University, Melbourne, Victoria, Australia.

4) City of Vantaa, Finland

5) Faculty of Social Sciences, University of Helsinki, Helsinki, Finland

6) Research Institute of Psychology, SWPS University of Social Sciences and Humanities, Wroclaw, Poland

7) Administrative Data Research Centre - Northern Ireland (ADRC-NI), Queen’s University Belfast, Belfast, UK

8) Finnish Institute for Health and Welfare, Helsinki, Finland

**Online Resource Table 1: Mean and standard deviations of SA episodes by recurrent pain status (all observations*)**

|  | **All spells** | | | **Short episodes** | | | **Medium episodes** | | | **Long episodes** | | |
| --- | --- | --- | --- | --- | --- | --- | --- | --- | --- | --- | --- | --- |
|  | Mean | SD | Sum of episodes** | Mean | SD | Sum of episodes | Mean | SD | Sum of episodes | Mean | SD | Sum of episodes |
| **All** |  |  |  |  |  |  |  |  |  |  |  |  |
|  | 7.5 | 8.3 | 38170 | 5.1 | 6.1 | 25862 | 1.8 | 2.6 | 9155 | 0.6 | 1.1 | 3153 |
| **Longitudinal multisite pain profiles** |  |  |  |  |  |  |  |  |  |  |  |  |
| No past or current pain | 5.6 | 6.6 | 12987 | 3.9 | 4.9 | 9180 | 1.3 | 2.1 | 2922 | 0.4 | 0.9 | 885 |
| Only current single-site pain | 7.4 | 7.5 | 3533 | 4.9 | 5.2 | 2341 | 1.8 | 2.7 | 881 | 0.6 | 1.1 | 311 |
| Only current multisite pain | 9.6 | 10.4 | 3770 | 6.4 | 7.7 | 2539 | 2.2 | 3.2 | 885 | 0.9 | 1.3 | 346 |
| Only past single-site pain | 7.3 | 8.5 | 3605 | 5.2 | 6.8 | 2543 | 1.6 | 2.2 | 805 | 0.5 | 0.9 | 257 |
| Recurrent single-site pain | 7.3 | 8.3 | 1853 | 4.9 | 6.3 | 1237 | 1.7 | 2.4 | 446 | 0.7 | 1.2 | 170 |
| Past single-site, current multisite pain | 9.6 | 9.5 | 3066 | 6.0 | 6.7 | 1912 | 2.6 | 3.1 | 826 | 1.0 | 1.6 | 328 |
| Only past multisite pain | 7.5 | 8.0 | 2000 | 5.2 | 6.4 | 1397 | 1.7 | 2.4 | 465 | 0.5 | 0.9 | 138 |
| Past multisite pain, current single-site | 8.7 | 10.8 | 1965 | 5.8 | 7.9 | 1320 | 2.1 | 3.0 | 477 | 0.7 | 1.3 | 168 |
| Recurrent multisite pain | 10.0 | 9.9 | 5391 | 6.3 | 7.3 | 3393 | 2.7 | 3.2 | 1448 | 1.0 | 1.5 | 550 |
| *Same person is possibly twice in the data **Total number of episodes in the data | | | | | | | | | | | | |

**Online Resource Table 2: Person-years, number of DP events and rates per 1000 person-years**

|  | **Person-years** | **Disability pension events** | **Rate per 1000 person years** |
| --- | --- | --- | --- |
|  |  |  |  |
| No past or current pain | 8905 | 46 | 5.2 |
| Only current single-site pain | 1870 | 17 | 9.1 |
| Only current multisite pain | 1563 | 38 | 24.3 |
| Only past single-site pain | 1922 | 14 | 7.3 |
| Recurrent single-site pain | 957 | 20 | 20.9 |
| Past single-site, current multisite pain | 1268 | 34 | 26.8 |
| Only past multisite pain | 1004 | 7 | 7 |
| Past multisite pain, current single-site pain | 833 | 15 | 18 |
| Recurrent multisite pain | 1821 | 67 | 36.8 |
|  |  |  |  |
| Total | 20143 | 258 | 12.8 |

**Online Resource Table 3: Current pain areas of the longitudinal pain groups in panel 1 (2007)**

|  | | **Longitudinal pain groups** | | | | | |  |  |  |
| --- | --- | --- | --- | --- | --- | --- | --- | --- | --- | --- |
|  | | **Only current single-site pain** | **Only current multisite pain** | **Recurrent single-site pain** | **Past single-site, current multisite pain** | **Past multisite pain, current single-site** | **Recurrent multisite pain** | **Total** |  |  |
| Pain location in 2007 | | % reporting | % | % | % | % | % | N |  |  |
| Pain in head or face | | 1 | 15 | 2 | 18 | 3 | 27 | 166 |  |  |
| Pain in neck or shoulder | | 25 | 67 | 32 | 71 | 28 | 84 | 731 |  |  |
| Pain in low back | | 16 | 50 | 12 | 53 | 13 | 62 | 509 |  |  |
| Pain in upper limbs | | 17 | 43 | 13 | 42 | 15 | 55 | 447 |  |  |
| Pain in lower limbs | | 30 | 50 | 31 | 51 | 31 | 61 | 589 |  |  |
| Pain in stomach location | | 2 | 10 | 2 | 19 | 6 | 16 | 135 |  |  |
| Pain in some other location | | 10 | 11 | 8 | 11 | 6 | 8 | 125 |  |  |
|  | The Helsinki Health Study. % does not sum up to 100 in profiles with multisite pain. | | | | | | | |  |  |

**Online Resource Table 4: Past pain areas of the longitudinal pain groups in panel 1 (2007)**

|  | **Longitudinal pain groups** | | | | | |  |
| --- | --- | --- | --- | --- | --- | --- | --- |
|  | **Only past single-site pain** | **Recurrent single-site pain** | **Past single-site, current multisite pain** | **Only past multisite pain** | **Past multisite pain, current single-site** | **Recurrent multisite pain** | **Total** |
| Pain location in 2000/2 | % reporting | % | % | % | % | % | N |
| Pain in head or face | 4 | 5 | 3 | 17 | 22 | 25 | 148 |
| Pain in neck or shoulder | 43 | 42 | 41 | 77 | 72 | 84 | 726 |
| Pain in low back | 13 | 10 | 18 | 41 | 52 | 50 | 359 |
| Pain in upper limbs | 11 | 8 | 11 | 42 | 39 | 54 | 331 |
| Pain in lower limbs | 17 | 27 | 17 | 36 | 40 | 45 | 360 |
| Pain in stomach location | 5 | 2 | 5 | 11 | 12 | 20 | 116 |
| Pain in some other location | 7 | 6 | 5 | 11 | 10 | 10 | 98 |

The Helsinki Health Study. % does not sum to 100 in profiles with multisite pain.

**
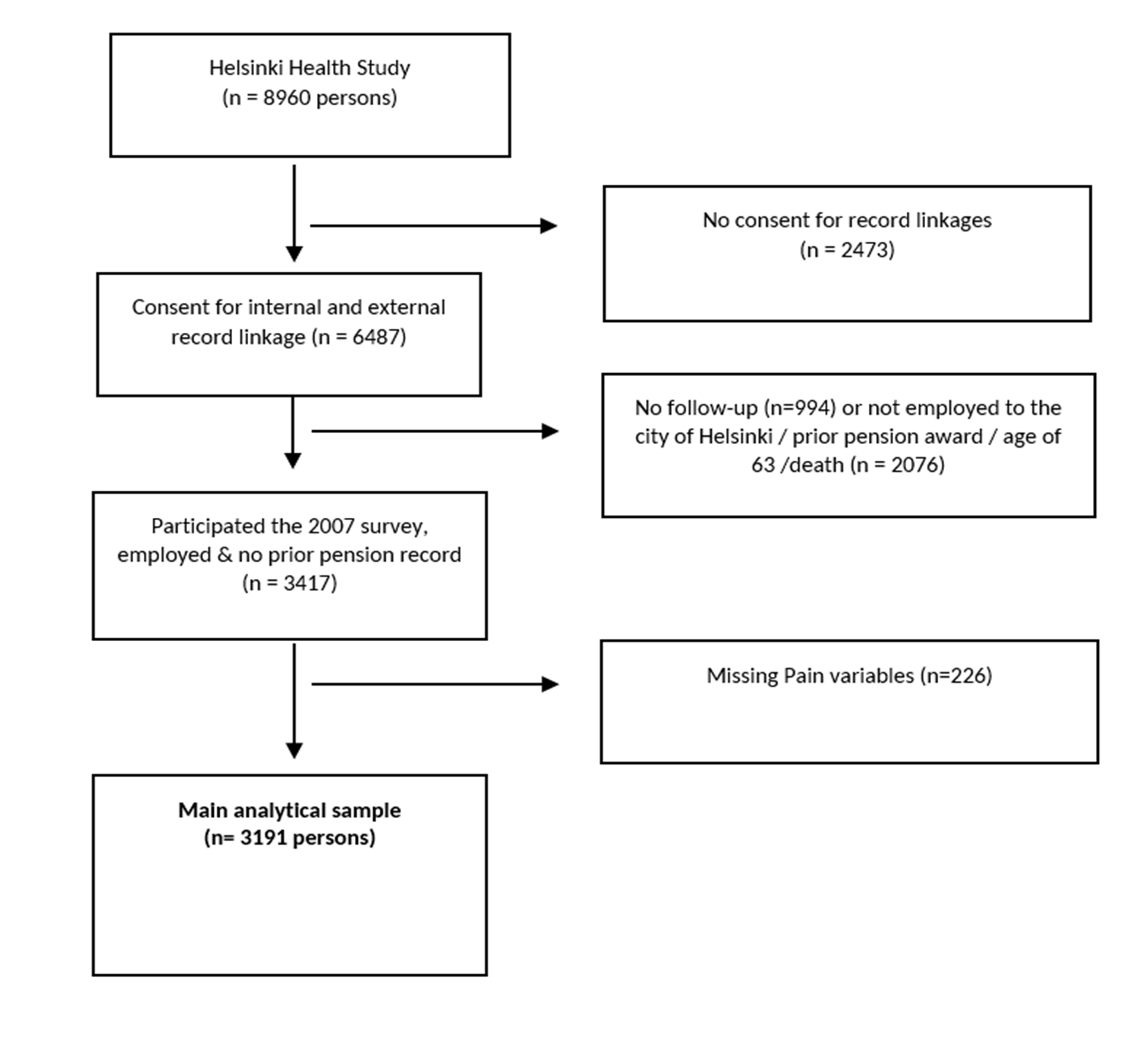
**

**Online Resource Figure 1** Flowchart of the study population

**
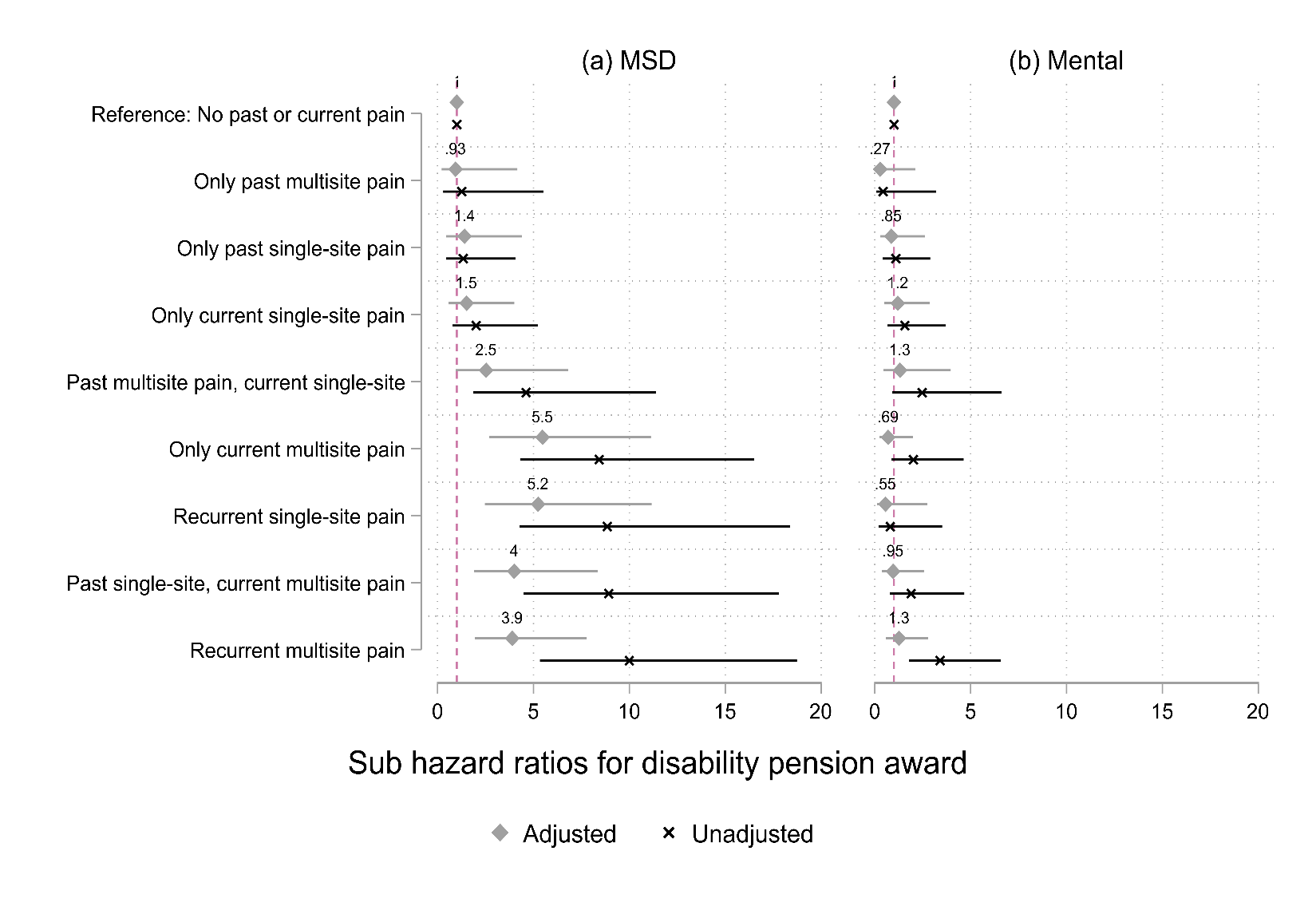
**

**Online Resource Figure 2** Sub hazard ratios for the association between types of recurrent pain and disability pension award due to musculoskeletal diseases (M00-99) and mental disorders (F00-99). Reference: no past or current pain.

1. Unadjusted model =adjusted for gender, measurement period
2. Adjusted model = additionally adjusted for occupational social class, long-standing illness, pain-related illness, working conditions, part-time work, marital status, common mental disorders, shift work and obesity
